# Isolated schwannoma originating from the infraorbital nerve: A case report

**DOI:** 10.1002/ccr3.6458

**Published:** 2022-11-27

**Authors:** Mahan Shafie, Mahsa Mayeli, Mohammad Taher Rajabi

**Affiliations:** ^1^ School of Medicine Tehran University of Medical Sciences Tehran Iran; ^2^ Iranian Center of Neurological Research, Neuroscience Institute Tehran University of Medical Sciences Tehran Iran; ^3^ NeuroTRACT Association, Students' Scientific Research Center Tehran University of Medical Sciences Tehran Iran; ^4^ Department of Ophthalmology Farabi Eye Hospital Tehran Iran

**Keywords:** infraorbital nerve, orbit, Schwannoma

## Abstract

As a benign isolated and encapsulated tumor originating from the nerve sheaths, schwannomas rarely appear on the cranial nerves, among which infraorbital nerve schwannomas are highly uncommon. We report a case of isolated infraorbital nerve schwannoma in a 58‐year‐old male patient with a clinical manifestation of proptosis in the affected eye.

## Abstract 2

As a benign tumor, schwannomas very rarely were reported to originate from the infraorbital nerve. Considering the reports of this tumor, diagnostic considerations when visiting patients with cystic masses in the infraorbital region are recommended.

## 1 INTRODUCTION

Schwannomas are benign, encapsulated, slow‐growing nerve sheath tumors that originate from any myelinated peripheral or central nerve.^1^ 1%–8% of all head and neck tumors are diagnosed as schwannomas, and also, orbital schwannomas are rare and account for 1%–4% of all orbital tumors.^2^ They are usually solitary with well‐defined margins and usually presented with slow progressive and painless mass, which can cause ocular proptosis or push of the eyeball.^3^ In the early stages, schwannoma can be asymptomatic; however, as the tumor grows, it may be symptomatic due to compression of the nerve or the adjacent structure. Orbital schwannomas more frequently arise from supra‐orbital and supra‐trochlear nerve, and infraorbital nerve involvement seems rare.^1^ In this study, we report a rare, benign, and isolated case of schwannoma arising from the infraorbital nerve in a case who initially presented to our center with proptosis and suborbital mass.

## 2 CASE PRESENTATION

**FIGURE 1:**
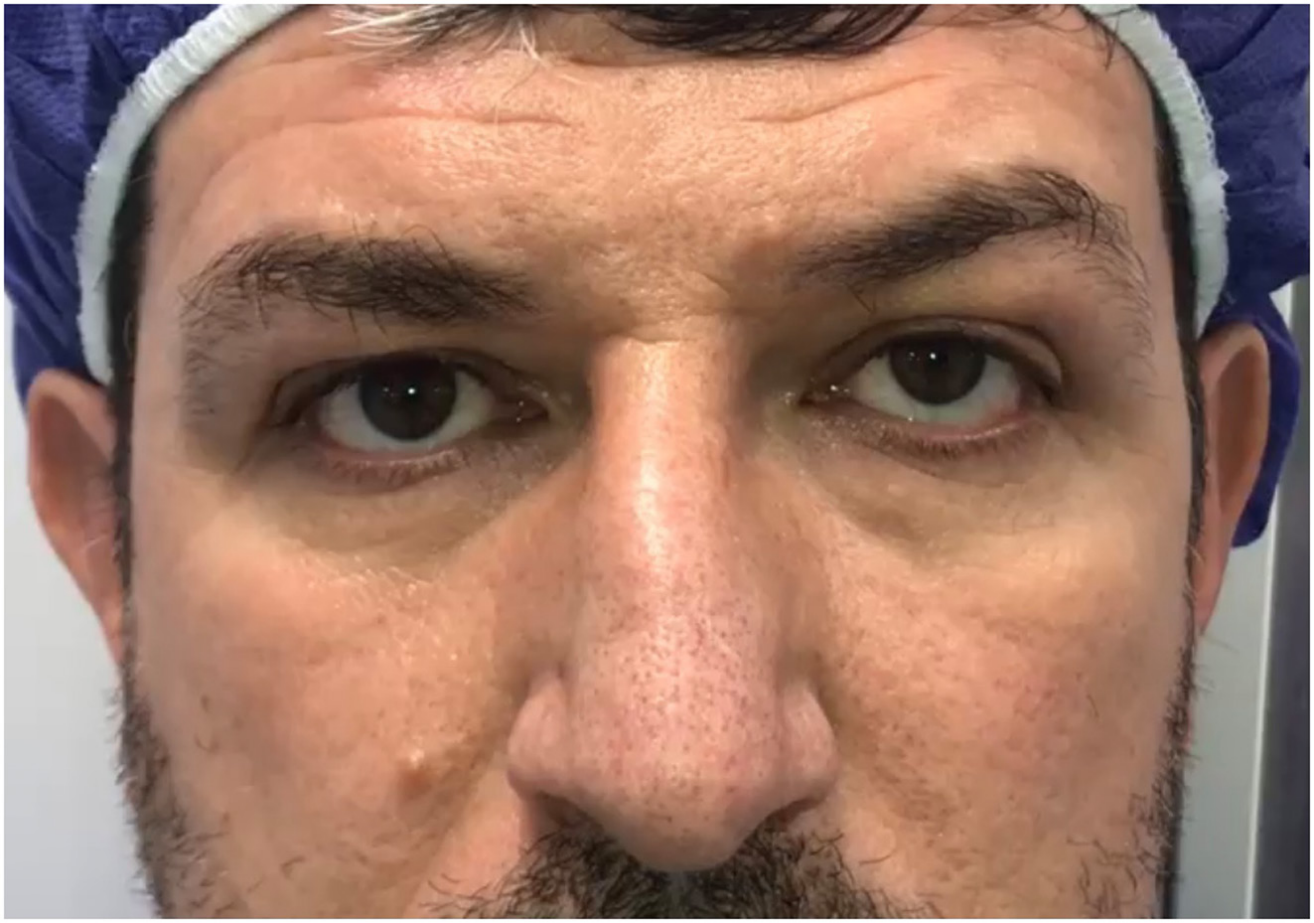
Superior displacement of the left eye

**FIGURE 2:**
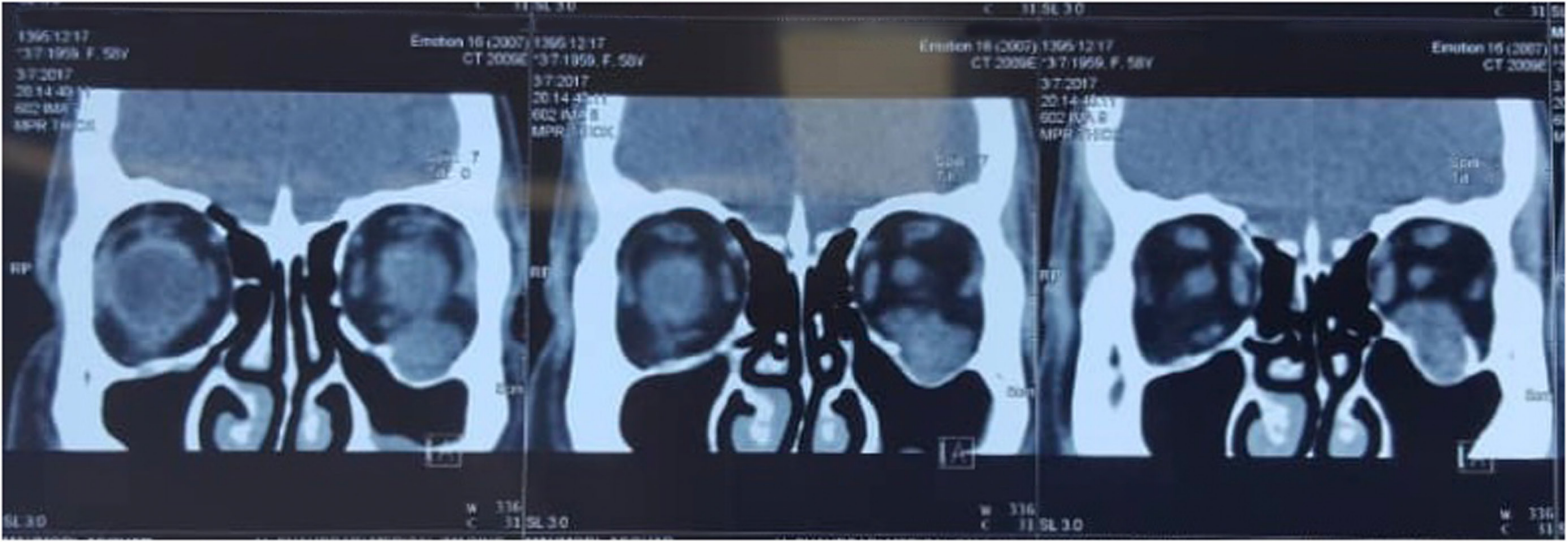
CT showed infraorbital canal enlargement due to homogenous, non‐calcified mass on the left side of coronal section. The posterior wall bowed into the maxillary sinus and the anterior wall rotated to the orbit globe.

**FIGURE 3:**
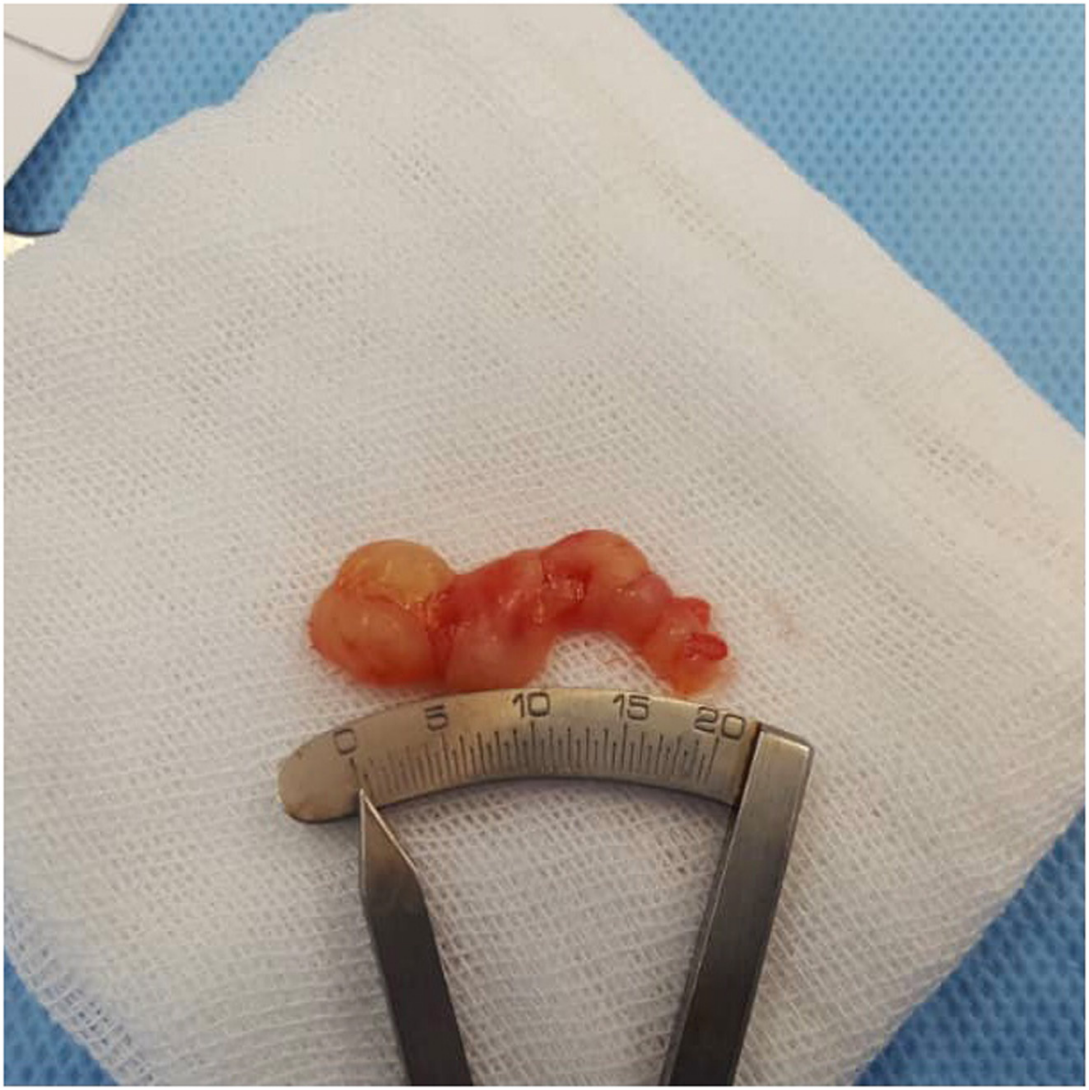
Smooth well‐encapsulated solid 8 × 20 mm mass

**FIGURE 4:**
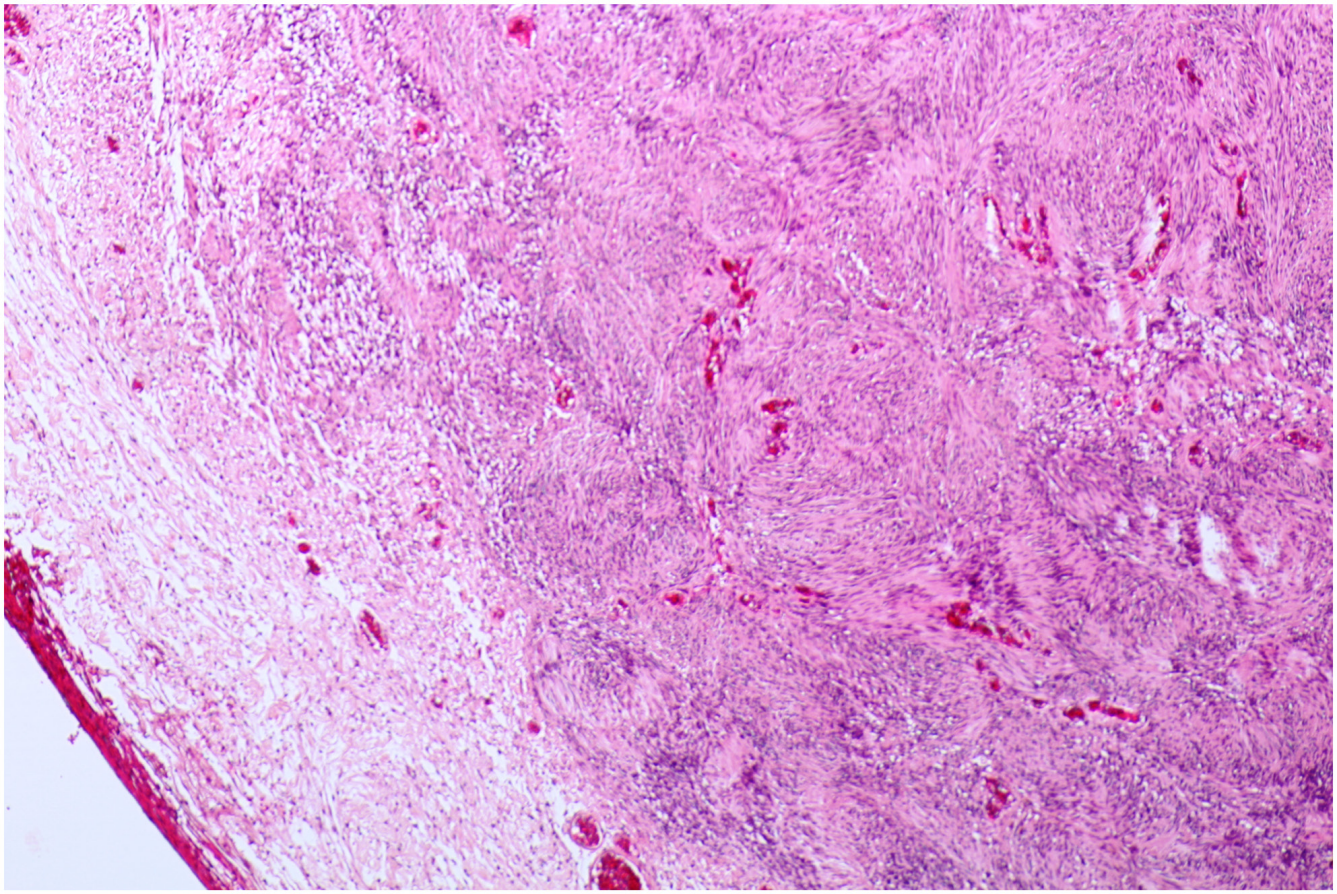
Patient's histopathology. Encapsulated biphasic spindle cell neoplasm (Schwannoma), H&E stain ×100

A 58‐year‐old man presented with a painless mass in the infraorbital region increasing in size over about past 6 months. The patient did not report a history of previous trauma or any other significant medical history. There was no family history of any significant illnesses. A firm, localized, non‐tender, mobile, subcutaneous round mass of about 2 cm was seen in the left infraorbital region on clinical examination. The skin, which was located on the mass, was normal, without any lesion or inflammation. The visual acuity, visual field, and color vision were normal. Eye movements were normal and lack of restriction and pain. He had 5 mm axial proptosis measured by Hertel exophthalmometry in addition to around 4 mm superior displacement on the globe mentioned as dystopia (Figure 1). The pupillary reactions were normal and supraorbital, infraorbital, and corneal sensations were intact. The cranial nerves and neurological examination were normal. The examination of slit‐lamp and fundoscopy also revealed normal findings in anterior and posterior segments. A computed tomography (CT) showed infraorbital canal enlargement due to homogenous, non‐calcified mass on the left side of the coronal section. The posterior wall bowed into the maxillary sinus and the anterior wall rotated to the orbit globe (Figure 2). The patient also underwent magnetic resonance imaging (MRI), and the well‐defined and non‐invasive mass were detected in the infraorbital region. On T1‐weighted MRI, homogenous, hypointense mass was observed and on T2‐weighted MRI, the mass was presented as hyperintense mass. Due to cystic changes, no significant enhancement was seen after gadolinium injection. Eventually, a surgical approach was applied to remove this tumor. After transconjunctival approaching to infraorbital region and dissection, orbital rim was achieved, and cystic mass was exposed. Afterward, dissection within the infraorbital canal was performed and the mass was removed as much as possible. After surgical removal, pathology reported a smooth well‐encapsulated solid 8 × 20 mm mass (Figure 3) consistent with schwannoma histopathology (Figure 4). The patient's condition improved well postoperatively with no specific complications. CT scan on a day after surgery showed no bleeding and no sign of residual mass, and the patient was able to leave the hospital 2 days after surgery with no complaint. After 2 years of follow‐up, he just had a complaint of mild anesthesia in the left maxillary and the left lateral nasal area with no clinical evidence of recurrence.

## 3 DISCUSSION

Schwannomas are well‐differentiated, encapsulated, benign tumors arising from the Schwann cells of peripheral neural sheaths and can appear on any myelinated sheets. However, these tumors very rarely were reported to originate from orbital nerves and in particular infraorbital nerves. Altogether, orbital schwannomas represent 1%–4% of orbital tumors and usually arise from the supraorbital or supratrochlear nerves.^2^ Schwannomas originating from the infraorbital nerve were merely reported in a few cases based on the current literature.^4^ The potential differential diagnoses for an infraorbital schwannoma are neurofibroma, perineural tumor infiltration, and solitary fibrous tumor, which can be differentiated based on imaging and pathologic findings.^1^

Schwannomas generally manifest as solitary encapsulated peripheral nerve tumors with the age predisposition of the forties and are more prevalent in males^5^; however, one case was reported in a 14‐year‐old boy that was resected applying an osteoplastic maxillotomy.^6^ Although ocular lesions and involvement of the osseous skeleton could be observed in schwannoma of the infraorbital nerve region, due to the encapsulated and slow‐growing nature of these tumors, radical clinical manifestations are substantially rare.^7^ The other previous reported cases mentioned no remarkable clinical manifestations, except for one, reporting localized paresthesia.^7^ That is contrary to our case, who experienced dystopia, proptosis, and facial disfiguration. Typically, the tumor originates from branches of either the supraorbital or supratrochlear nerves and therefore, downward displacement of the globe is observed; however, less commonly, it may arise from the infraorbital nerve and produce upward displacement.^8^

Regarding the diagnostic approach, all aspects need to be considered to specify the diagnosis, including thorough clinical history, physical examination, imaging modalities, and histopathological examination. Former works suggested that cystic mass that is captured as a partial high‐density enhancement within the cyst in the ocular computerized tomography and the signal findings observed on MRI of a schwannoma, has been characterized as hypo or isointense signals on T1, homogeneous hyperintense signals on T2, all suggestive of a slow‐growing benign process. If neoplasia is suspected, the use of preoperative imaging is important to help distinguishing between benign and malignant lesions. Our findings were congruent with these results.^9^

To prevent the compression of the optic nerve, surgical excision is the treatment of choice and the tumor should be removed intact at the earliest. This recommendation is based on the fact that most schwannomas are progressively growing and might lead to compression of the nerve and possible nerve atrophy. This is to an extent that incomplete excision can cause recurrence or even intracranial extension. Additionally, these high cellular tumors have a chance of recurrence and malignant transformation, and early treatment are therefore recommended to avoid the complications related to progressive growth of the tumor.^8^ There was no evidence of recurrence and other complications after 2 years of follow‐up in our case.

Herein, a rare case of isolated infraorbital schwannoma presenting as a painless mass with no disturbance of visual acuity, merely manifesting a proptosis and dystopia in the left eye was introduced that was completely excised using a transconjunctival inferior orbitotomy approach. Considering the rarity of infraorbital schwannoma, diagnostic considerations for schwannoma as a possible diagnosis when visiting patients with infraorbital cystic masses are recommended. Imaging modalities including CT and MRI are recommended to make the diagnosis at this unusual location for schwannoma, and we also suggest a transconjunctival inferior orbitotomy surgical approach to enhance the cosmetic effects.

## AUTHOR CONTRIBUTIONS

The manuscript has been read and approved by all the authors. MS contributed to developing the research idea and design and composing and revising the manuscript. MM contributed to composing and revising the manuscript. MR contributed to developing the research idea and revising the manuscript.

## CONFLICT OF INTEREST

The authors have no conflict of interest to declare.

## ETHICAL APPROVAL

This study was approved by the research and ethics committee of Tehran University of Medical Sciences. The patient has given his informed consent to publish this case.

## CONSENT

Written informed consent was obtained from the patient for publication of this case report and any accompanying images. A copy of the written consent is available for review by the Editor‐in‐Chief of this journal.

## Data Availability

Data sharing is not applicable to this article as no datasets were generated or analyzed during the current study.
